# Role of Lifeguard β-isoform in the development of breast cancer

**DOI:** 10.3892/or.2014.3363

**Published:** 2014-07-25

**Authors:** NADJIB DASTAGIR, ANDREA LAZARIDIS, KHALED DASTAGIR, KERSTIN REIMERS, PETER M. VOGT, VESNA BUCAN

**Affiliations:** Department of Plastic, Hand and Reconstructive Surgery, Hannover Medical School, D-30659 Hannover, Germany

**Keywords:** Lifeguard, Lifeguard β-isoform, apoptosis, breast cancer

## Abstract

In the last century there has been great progress in the treatment of breast cancer by improving drug and radiation therapy as well as surgical techniques. Despite this development, breast cancer remains a major cause of death among women in Europe and the US. The cause of breast cancer at the cellular level is still not fully understood. In the present study, we investigated the expression of the Lifeguard β-isoform in breast cancer tissues. In contrast to Lifeguard, the β-isoform has one transmembrane domain less, which is the last of seven (99 bp), and due to this we suspect that the Lifeguard β-isoform exhibits a different function. We determined the expression and function of the β-isoform of Lifeguard in breast cancer cell lines (MCF-7 and MDA-MB-231), a human breast epithelial cell line (MCF10A) and in breast tumour tissue sections. Western blotting, PCR arrays and immunofluorescence were used to investigate the expression of Lifeguard and its β-isoform. Moreover, we investigated the ability of Lifeguard β-isoform expression to inhibit apoptosis induced by Fas. Our results indicated that Lifeguard β-isoform is strongly expressed in breast tumour tissues. More notably, we demonstrated that Fas sensitivity was reduced in the MCF10A breast cells expressing the Lifeguard β-isoform. Taken together, our findings indicate the role of the Lifeguard β-isoform as an anti-apoptotic protein and provide further evidence of the potential of the Lifeguard β-isoform as a target for the development of novel therapeutic strategies.

## Introduction

In the present study, we focused on the β-isoform of Lifeguard. This transmembrane protein belongs to the uncharacterised protein family UPF0005. The long version termed Lifeguard has already been identified as a molecule that inhibits death mediated by Fas in tumour cells (1–3). Although the mechanism and action of Lifeguard remain unclear, it is hypothesised that it either plays a role by interacting with Fas or at the level of the Fas/FADD complex. Nevertheless, it was previously shown that Lifeguard interacts with Bax and is localised in cellular membranes of the endoplasmic reticulum and in the plasma membrane (3–5). Previously, Lifeguard was found to be highly expressed in breast carcinoma tissues. The expression of Lifeguard was found to correlate with high tumour grades in primary breast tumours and it was dependent on the activity of Akt/LEF-1 signalling (6).

To date, there are no published data evaluating the role of the Lifeguard β-isoform in carcinogenesis. In the present study we eavaluated the expression of Lifeguard β-isoform in breast cancer cell lines *in vitro* and its expression in human breast cancer tissue samples by western blotting and immunofluorescence. We tested the functional relationship between Fas and Lifeguard β-isoform expression by demonstrating the correlation between Lifeguard β-isoform expression and resistance against cell death stimulation by an agonist Fas antibody.

## Materials and methods

### Cell lines

A normal human mammary cell line (MCF10A), derived from normal breast epithelium, three human breast carcinoma cell lines (MCF-7, MDA-MB-231, T47D) and a liposarcoma carcinoma U2OS cell line were used in this study. All of the cell lines were obtained from the American Type Culture Collection (ATCC; Manassas, VA, USA). MCF-7, MDA-MB-231 and U2OS cell lines were grown in Dulbecco’s modified Eagle’s medium (DMEM; PAA, Cölbe, Germany) supplemented with 10% FCS (Biochrom, Berlin, Germany) and 50 mg/ml penicillin-streptomycin. T47D cells were grown in RPMI-1640 medium with 0.2 U/ml bovine insulin (10 mg/ml). The MCF-10A cells were cultured in defined mammary epithelial growth media (CC-2571; MEGM Bullet Kit; Lonza, Inc.). All of the cells were maintained at 37°C in 5% carbon dioxide in a humidified atmosphere. The cells were subcultured every 2 to 3 days by treatment with 0.25% trypsin/0.53 mM ethylenediaminetetraacetic acid (EDTA) solution. Primary human breast cancer-derived epithelial cells (HBCECs) were obtained from explant cultures of human breast cancer biopsies after negative testing for HIV-1, hepatitis B and C, bacteria, yeast and fungi, respectively, as previously described (34). Informed written consent was obtained from each patient for the use of individual biopsy material, and the study was approved by the Institutional Review Board, Project #3916 on June 15, 2005. The primary HBCECs were cultured further in serum-free and phenol red-free mammary epithelial cell growth medium (MEGM) (Lonza, Basel, Switzerland) in a humidified atmosphere at 37°C. Half of the cell culture medium was replaced approximately every fourth day to maintain a conditioned medium.

### cDNA plate array

cDNA plate array analysis is a plate-based hybridization profiling technique that is used for monitoring the expression of dozens of genes through reverse transcription of mRNA into cDNA. For this analysis, total RNA was isolated from cells transfected with pMK-Lifeguard or with pMK-Lifeguard β-isoform vector (GeneArt, Regensburg, DE, USA), using the NucleoSpin RNA II kit (Macherey-Nagel, Düren, Germany). RNA samples (8 μg) were analyzed by microarray analysis using an Akt pathway-regulated cDNA plate array (Signosis, Sunnyvale, CA, USA) according to the manufacturer’s instructions. Each well on the plate contained a cDNA probe for one of the 24 Akt pathway-regulated genes. After reverse transcription, *in situ* hybridisation, blocking and extensive washing, the wells were incubated with streptavidin-HRP, and the resulting chemiluminescence was measured within 5 min using a luminometer (Tecan Schweiz AB, Zurich, Switzerland).

### Western blot analysis

For the western blot analysis, the cells were lysed in RIPA buffer containing 0.3 M NaCl, 1% sodium desoxycholate, 0.1% sodium dodecyl sulfate (SDS), 1% Triton X-100, 20 mM Tris-HCL (pH 8.0), 1 mM EDTA and 1 mM phenylmethylsulfonyl fluoride (PMSF). Protein (25 μg) was fractionated by 15% SDS-PAGE and transferred to polyvinylidene fluoride (PVDF) membranes (Millipore Corporation, Bedford, MA, USA). The membranes were blocked in Odyssey buffer (Li-COR Biosciences, Lincoln, NE, USA) for 1 h. The protein expression levels were determined by immunoblotting with the polyclonal antibodies anti-hLifeguard β-isoform (1:200 dilution; generated in our laboratory) and monoclonal anti-hNGF-R, TrK-A/B, Act-p, actin (all used at 1:500; Abcam, Cambridge, UK) at 4°C overnight. To quantify the protein expression levels, Odyssey 680/800 nm secondary conjugates were used, and the PVDF membranes were analyzed using the Odyssey infrared imaging system and software (Li-COR Biosciences).

### Immunofluorescence

Breast tissue slides were deparaffinised in xylene followed by an alcohol gradient. To reduce the non-specific background staining, the slides were incubated in 0.3% bovine serum albumin/1X Tris-buffered saline for 30 min and incubated with hLifeguard-β-iso rabbit primary antibodies at 4°C overnight. The slides were washed twice for 5 min with phosphate-buffered saline (PBS) and incubated for 30 min with goat anti-rabbit Alexa Fluor 488 (Invitrogen) secondary antibody. The signals were detected using the Axiovert 200M fluorescence microscope (Zeiss) equipped with the appropriate barrier filters.

### Caspase-3/7 assay

Activation of caspase-3/7 was determined using the Apo-One Homogeneous Caspase-3/7 assay (Promega, Madison, WI, USA) according to the manufacturer’s instructions. Briefly, MCF-10A breast cells were seeded (1×10^4^/well) in a 96-well plate and transfected with pMK-Lifeguard and pMK-Lifeguard β-isoform vector for 24 h. After 24 h, the cells were incubated with 50 ng/ml agonistic anti-Fas (clone CH11; Abcam) for an additional 24 h. Following treatment, the cells were mixed with the same volume of Apo-One Homogeneous Caspase-3/7 reagent and incubated at room temperature for 2 h. Caspase-3/7 activation was estimated from sample fluorescence at the excitation wavelength of 492 nm and the emission wavelength of 521 nm using the fluorescence plate reader Tecan GENios (Tecan Schweiz AB, Zurich, Switzerland).

## Results

### Expression of Lifeguard β-isoform in human breast cancer cells and tissues

To elucidate the role of the Lifeguard β-isoform in the regulation of apoptosis in breast cancer, we first assessed the protein expression of the Lifeguard β-isoform in different human breast cancer cells and tissues. The human Lifeguard β-isoform was observed only in the patient primary cell cultures from invasive breast carcinoma with tumour grade III (cells appeared abnormal and tended to grow and spread more aggressively), but was not detected in the breast cancer cell lines and the liposarcoma carcinoma U2OS cell line (Fig. 2A).

To test the relevance of Lifeguard β-isoform protein expression in patient primary breast carcinoma cells derived from human breast cancer specimens, we examined the expression of Lifeguard β-isoform protein in carcinoma breast tissue sections. Representative image pairs, detected by microscopy from tissue samples with different tumour grades with varying levels of the Lifeguard β-isoform protein expression compared with the control staining, are shown in Fig. 2B.

### Overexpression of the Lifeguard β-isoform inhibits apoptosis and induces the expression of genes of the Akt2 pathway

**Figure 1:**
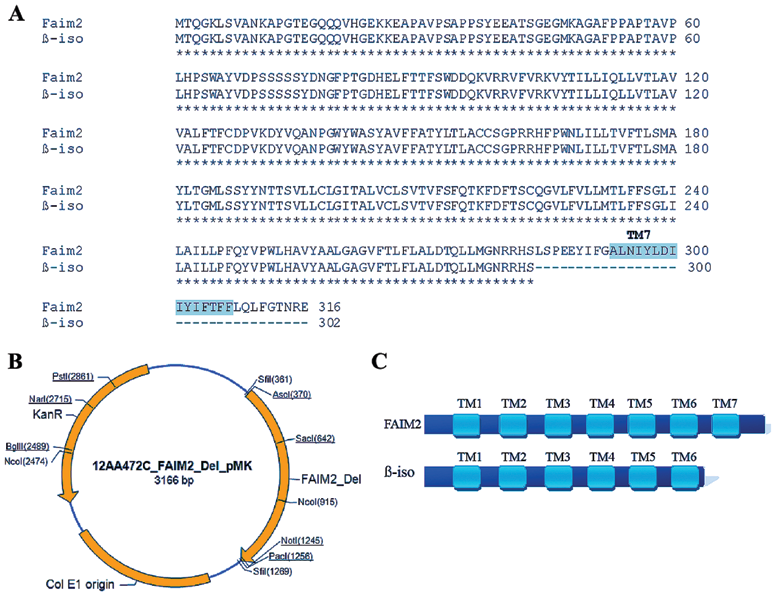
Schematic illustration of Lifeguard and Lifeguard β-isoform sequences. (A and C) Compared to Lifeguard, the β-isoform consists of a stop codon in position 302 resulting in a shorter polypeptide; the sequence NP_036438 (amino acids ALNIYLDIIYIFTFF), which represents the seventh transmembrane (TM) domain shaded in blue is missing in the β-isoform. (B) Schematic illustration of the modified pMK-KmR vector for Lifeguard/Lifeguard β-isoform cloning.

To investigate the ability of Lifeguard β-isoform expression to suppress Fas-induced apoptosis, the human breast cell line MCF10A without endogenous Lifeguard β-isoform expression was selected. Two vectors were designed, pMK-Lifeguard and pMK-Lifeguard β-isoform (Fig. 1A and B) and were tested for their activity (data not shown). The MCF10A cells were transfected with pMK-Lifeguard β-isoform for 24 h and treated with 50 ng/ml of agonistic anti-Fas. Following 24 h of incubation, significantly increased levels of caspase 3/7 were detected in the non-transfected cells when compared to the levels in the Lifeguard and Lifeguard β-isoform transfectants (Fig. 3A). The inactivation of apoptosis revealed the Lifeguard β-isoform to be a potential regulator of apoptosis in tumour cells.

Taking into consideration the anti-apoptotic activity of Lifeguard β-isoform, we next aimed to ascertain the genes that are expressed at higher levels when the β-isoform is present by analysis using the human Akt pathway regulated cDNA plate array kit. Before RNA isolation, the MCF10A cells were transfected with pMK-Lifeguard or pMK-Lifeguard β-isoform, respectively, for 24 h. The results showed that the Lifeguard β-isoform-transfected cells exhibited upregulated expression of 4E-BP and p27 expression as well as the expression of Akt1 and Akt2 compared to the Lifeguard-transfected cells (Fig. 3B).

### Nerve growth factor-induced Lifeguard β-isoform expression

In order to demonstrate a direct effect of the nerve growth factor (NGF) on Lifeguard β-isoform expression, we treated the MCF10A breast cell line with 0.5 or 1.5 μg/ml NGF for 24 h. Analysis of cellular protein lysates from the MCF10A cells by western blotting demonstrated that NGF dose-dependently upregulated the expression of Lifeguard β-isoform protein. Furthermore, we found significant increases in the expression of TrK-A/B and Akt-p protein after treatment with NGF (Fig. 4A). These observations identify Lifeguard β-isoform as a target of the NGF pathway (Fig. 4B), a regulation which could play a role in breast tumour progression.

## Discussion

Dysregulation of apoptosis plays an important role in the pathogenesis of human cancers (7). Lifeguard, a member of a unique gene family with high structural similarity (8), was isolated and identified as a molecule that inhibits death mediated by Fas in tumour cells. Given the high structural similarity and phylogenetic relationships among the Lifeguard proteins, Hu *et al* reported that it is highly likely that all Lifeguard family members are in some way apoptosis modulating (8). Somia *et al* showed that Lifeguard binds directly to the Fas receptor but not to Fas adaptor proteins (1). The anti-apoptotic role of Lifeguard has been tested in LN-18 astrocytoma, cervical carcinoma HeLa and Jurkat T cell lines (10), yet the exact mechanism of action of Lifeguard remains unclear. It is well documented that dominant-negative Akt/PKB inhibits Lifeguard activity, whereas overexpression of constitutively active Akt/PKB increases Lifeguard activity (9,10). In comparison to Lifeguard, the β-isoform has one transmembrane domain less; specifically the last (seventh) transmembrane is missing. Due to this, we hypothesised that the Lifeguard β-isoform exhibits a different function (Fig. 1A and C).

**Figure 2:**
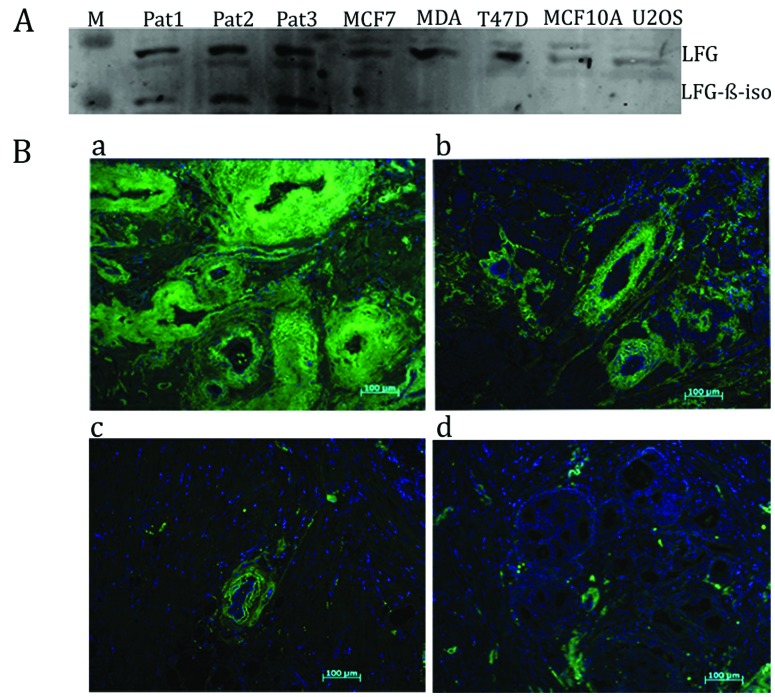
Expression of Lifeguard β-isoform. (A) Samples from three patient primary cell cultures from invasive breast carcinoma with tumour grade III (Pat1, Pat2 and Pat3), three human breast carcinoma cell lines [MCF-7, MDA-MB-231 (MDA) and T47D], a normal human mammary cell line (MCF10A) and a liposarcoma carcinoma U2OS cell line were analyzed by western blotting. Lifeguard β-isoform protein expression was detectable only in the primary cell cultures. (B) The lower the differentiation of the human breast cells, the stronger was the expression of Lifeguard β-isoform: (a) invasive ductal adenocarcinoma, poorly differentiated; (b) invasive ductal adenocarcinoma, moderately differentiated; (c) invasive ductal adenocarcinoma, well differentiated; (d) antibody control.

**Figure 3:**
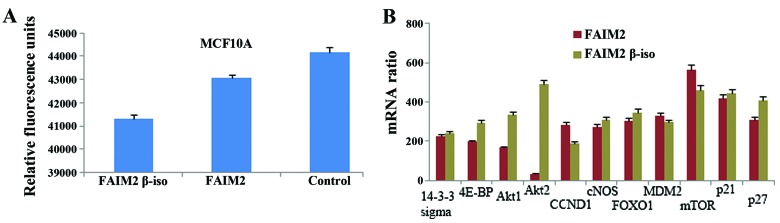
Influence of Lifeguard β-isoform. (A) Comparison of the sensitivity of Lifeguard compared to the Lifeguard β-isoform in affecting apoptosis induced by Fas stimuli in a normal breast cell line. (B) MCF10A cells were transfected with the vectors pKM-Lifeguard (red) or pKM-Lifeguard β-isoform (brown), and changes in gene expression were analysed using the Akt pathway regulated cDNA plate array.

In the present study, we examined the expression of Lifeguard β-isoform protein in normal breast and carcinoma cell lines and carcinoma tissues. We provide convincing evidence that expression of Lifeguard β-isoform protein was increased in breast carcinoma versus normal cells and tissues. Moreover, we found convincing evidence that the expression of the Lifeguard β-isoform protein was increased in breast carcinoma relative to differentiated tissues (Fig. 2B). In contrast to BI-1, for which high expression rates have been demonstrated in several tumour tissues and cancer cell lines (11–14) and Lifeguard (3) this is the first time that high Lifeguard β-isoform expression rates can be phenotypically linked to human cancer (4). Resistance to apoptosis and alterations in Fas signalling were initially observed in breast carcinoma cell lines (15). Several further studies on breast cancer patients indicated that the Fas/FasL status may have a significant impact on patient survival (16–19). These results, together with the evidence obtained during experiments on other solid malignancies (20–26), suggest that the tumour levels of Fas/FasL possibly influence the prognosis of oncology patients. In the present study, we found that Lifeguard β-isoform protein expression reduced the sensitivity against stimulation with an agonistic Fas antibody (Fig. 3A) even more than the long version of Lifeguard, which has been previously identified as a molecule that inhibits death mediated by Fas in tumour cells (8).

In regards to breast cancer, studies have focused on the opposing functions of Akt1 and Akt2 on cell migration and invasion. In this study, we found that the Akt2 isoform was activated to a greater degree by the Lifeguard β-isoform than by Lifeguard (Fig. 3B). Several downstream Akt targets have been shown to contribute to the differential functions of these two isoforms in motility, including palladin, nuclear factor of activated T cells, tuberous sclerosis complex 2 and β1 integrins (27–30).

**Figure 4:**
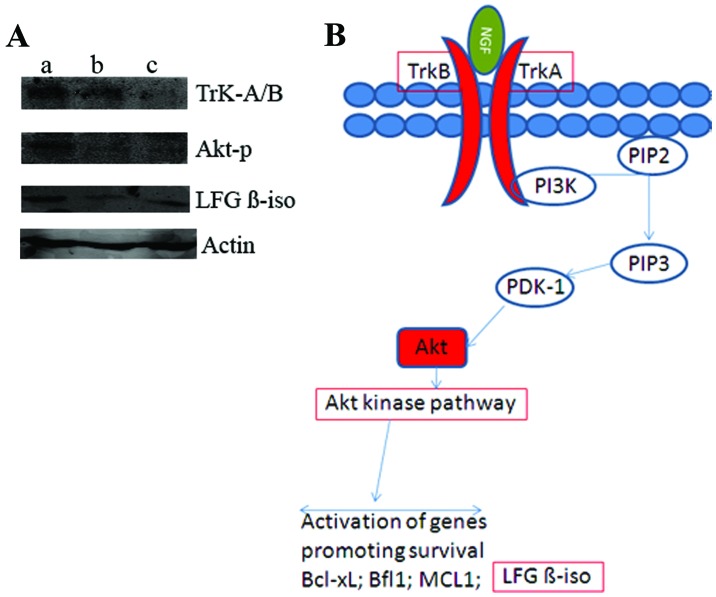
Nerve growth factor (NGF) induces expression of the Lifeguard (LFG) β-isoform. (A) Western blotting was used to demonstrate the expression of Lifeguard β-isoform, receptor tyrosine kinase A/B and phosphorylation of Akt after treatment with NGF: (a) 1.5 μg/ml NGF; (b) 0.5 μg/ml NGF; (c) control without NGF. (B) Lifeguard β-isoform may be induced by PI3-kinase-Akt/PKB signalling which can be activated by NGF.

Concerning breast cancer, it has been shown that NGF promotes both tumour cell survival and proliferation (31–33). In the present study, we first demonstrated that addition of NGF increased Lifeguard β-isoform activity in MCF10A breast cells (Fig. 4A). Furthermore, we determined that TrK-A/B and p-Akt protein expression levels were increased following exposure to NGF. These data suggest that over-expression of the Lifeguard β-isoform in breast cells has oncogenic potential.

In conclusion, in the present study we have shown that high levels of Lifeguard isoform expression are associated with the grade of the breast tumour. More notably, we found that Lifeguard β-isoform plays a greater role than Lifeguard in the development of breast cancer. Thus, the Lifeguard β-isoform is a potential target for therapeutic benefit in cancer.
